# Depicting the phenotypic space of the annual plant *Diplotaxis acris* in hyperarid deserts

**DOI:** 10.1002/ece3.8232

**Published:** 2021-11-05

**Authors:** Nasr H. Gomaa, F. Xavier Picó

**Affiliations:** ^1^ Department of Botany and Microbiology Faculty of Science Beni‐Suef University Beni‐Suef Egypt; ^2^ Biology Department College of Science Jouf University Sakaka Saudi Arabia; ^3^ Departamento de Ecología Integrativa, Estación Biológica de Doñana (EBD) Consejo Superior de Investigaciones Científicas (CSIC) Sevilla Spain

**Keywords:** Architectural traits, *Diplotaxis acris*, fitness, genetic variation, hyperarid deserts, integrated phenotype, life history traits, phenotypic space

## Abstract

The phenotypic space encompasses the assemblage of trait combinations yielding well‐suited integrated phenotypes. At the population level, understanding the phenotypic space structure requires the quantification of among‐ and within‐population variations in traits and the correlation pattern among them. Here, we studied the phenotypic space of the annual plant *Diplotaxis acris* occurring in hyperarid deserts. Given the advance of warming and aridity in vast regions occupied by drylands, *D. acris* can indicate the successful evolutionary trajectory that many other annual plant species may follow in expanding drylands. To this end, we conducted a greenhouse experiment with 176 *D. acris* individuals from five Saudi populations to quantify the genetic component of variation in architectural and life history traits. We found low among‐population divergence but high among‐individual variation in all traits. In addition, all traits showed a high degree of genetic determination in our study experimental conditions. We did not find significant effects of recruitment and fecundity on fitness. Finally, all architectural traits exhibited a strong correlation pattern among them, whereas for life history traits, only higher seed germination implied earlier flowering. Seed weight appeared to be an important trait in *D. acris* as individuals with heavier seeds tended to advance flowering and have a more vigorous branching pattern, which led to higher fecundity. Population divergence in *D. acris* might be constrained by the severity of the hyperarid environment, but populations maintain high among‐individual genetic variation in all traits. Furthermore, *D. acris* showed phenotypic integration for architectural traits and, to a lesser extent, for life history traits. Overall, we hypothesize that *D. acris* may be fine‐tuned to its demanding extreme environments. Evolutionary speaking, annual plants facing increasing warming, aridity, and environmental seasonality might modify their phenotypic spaces toward new phenotypic configurations strongly dominated by correlated architectural traits enhancing fecundity and seed‐related traits advancing flowering time.

## Graphical abstract

The phenotypic space encompasses the assemblage of trait combinations yielding well‐suited integrated phenotypes. We conducted a greenhouse experiment with 176 *Diplotaxis acris* individuals from five Saudi populations to quantify the genetic component of variation in architectural and lifehistory traits. Overall, we conclude that *D. acris* may exhibit fine‐tuned phenotypes in its demanding extreme environments.

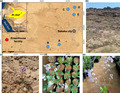

## 1 INTRODUCTION

Adaptive evolution is a pervasive process by which purifying selection eliminates the vast majority of deleterious mutations in a population, filtering out disadvantageous genetic variants and, in the end, unsuitable phenotypes. Over generations, a population molds its genetic composition and builds its phenotypic space (Fraebel et al., 2017; Murren, 2012; Pigliucci, 2007; Schlichting & Pigliucci, 1995), which encompasses the assemblage of trait combinations of well‐suited phenotypes. One way to understand how the phenotypic space is structured deals with the quantification of among‐ and within‐population genetic variations in phenotypic traits as well as the intrapopulation correlation pattern among traits (Benavides et al., 2021; Messier et al., 2018). At the population scale, correlations among traits mainly reflect genetic, developmental, or physiological processes (Armbruster et al., 2014; Messier et al., 2018). Broadly speaking, the interplay between genetic constraints, for example, pleiotropic interactions with potential antagonistic selection on correlated traits (Auge et al., 2019; Keith & Mitchell‐Olds, 2019), and natural selection represents the major force shaping the phenotypic space (Lande & Arnold, 1983; Messier et al., 2018; Pigliucci, 2007). Nevertheless, genetic constraints and natural selection may also act as a means to preserve successful trait combinations boosting the phenotypic space structure (Auge et al., 2019; Wagner et al., 2008).

Regardless of the several ways in which natural selection determines the distribution of phenotypes within a population, along with the omnipresent random effects of genetic drift, the severity of the environment eventually imposes the ecological limits of the phenotypic space. Field studies in plants revealed how environmental gradients (e.g., elevation and core–periphery clines) influenced mean trait values and also the pattern of trait covariation within populations (Benavides et al., 2021; Boucher et al., 2013; Dwyer & Laughlin, 2017; Michelaki et al., 2019; Rosas et al., 2019; Umaña & Swenson, 2019). Furthermore, some of these studies did show how the degree of phenotypic integration, given by the strength of covariation among traits, intensified with increasing environmental harshness (Dwyer & Laughlin, 2017; Michelaki et al., 2019; Benavides et al., 2021; but see Boucher et al., 2013). Although these field studies were not exclusively focused on life history traits and did not quantify the underlying genetic component of phenotypic integration, they shed light on the consequences of intraspecific trait variability and phenotypic integration in natural populations for plant community assembly and ecosystem functioning.

A deeper understanding of how organisms integrate their phenotypes in extreme environments may provide insights into the evolutionary pathways required to endure the current scenario of rapid increasing climate‐related risks and extreme events in several world regions. In this sense, desert annuals represent an appropriate system to study phenotypic integration because they have developed a strategy to face the environmental unpredictability associated with harsh environments. In particular, desert annuals commonly exhibit delayed germination, which buffers variation in reproduction success due to greater environmental risk (i.e., bed hedging; Clauss & Venable, 2000; Pake & Venable, 1996; Venable, 2007). However, beyond the association between the germination fraction and reproductive success, we know less about the phenotypic space in desert annuals in terms of among‐ and within‐population variations and intrapopulation correlation among traits. Given the important demographic role of the seed stage (e.g., seed dormancy in the soil seed bank and germination timing; Clauss & Venable, 2000; Adondakis & Venable, 2004; Volis et al., 2004; Gomaa, 2020), we expect seed‐related traits, such as seed weight or germination behavior, to play a central role in structuring the phenotypic space in desert annuals.

The main goal of this study was to characterize among‐ and within‐population genetic variations for phenotypic traits and for the correlation pattern among traits in the annual plant *Diplotaxis acris* (Forssk.) Boiss. (Brassicaceae). The species occurs in hyperarid deserts where natural selection exerts strong pressures on standing genetic variation and resulting phenotypic distribution. We undertook a greenhouse experiment to quantify among‐ and within‐population genetic variations in architectural and life history traits in 176 *D. acris* individuals from five populations from the Arabian Desert. We computed broad‐sense heritability values for all traits, which provides an estimation of all genetic contributions to within‐population phenotypic variation. We also evaluated the effects of selection on recruitment and flowering time, the two major fitness‐related developmental transitions in annuals, and computed correlations among traits to estimate the degree of phenotypic integration in *D. acris*. Given the generalized trend for increasing temperatures and aridity is several world regions, particularly since the turn of the 21st century across the approximately 41% of the terrestrial land surface characterized as drylands (Huang et al., 2016), hyperarid deserts can teach us important lessons on successful phenotypic space in plants in such unforgiving hot, dry, and markedly seasonal environments.

## 2 MATERIALS AND METHODS

### 2.1 Study area, species, and source populations

**FIGURE 1:**
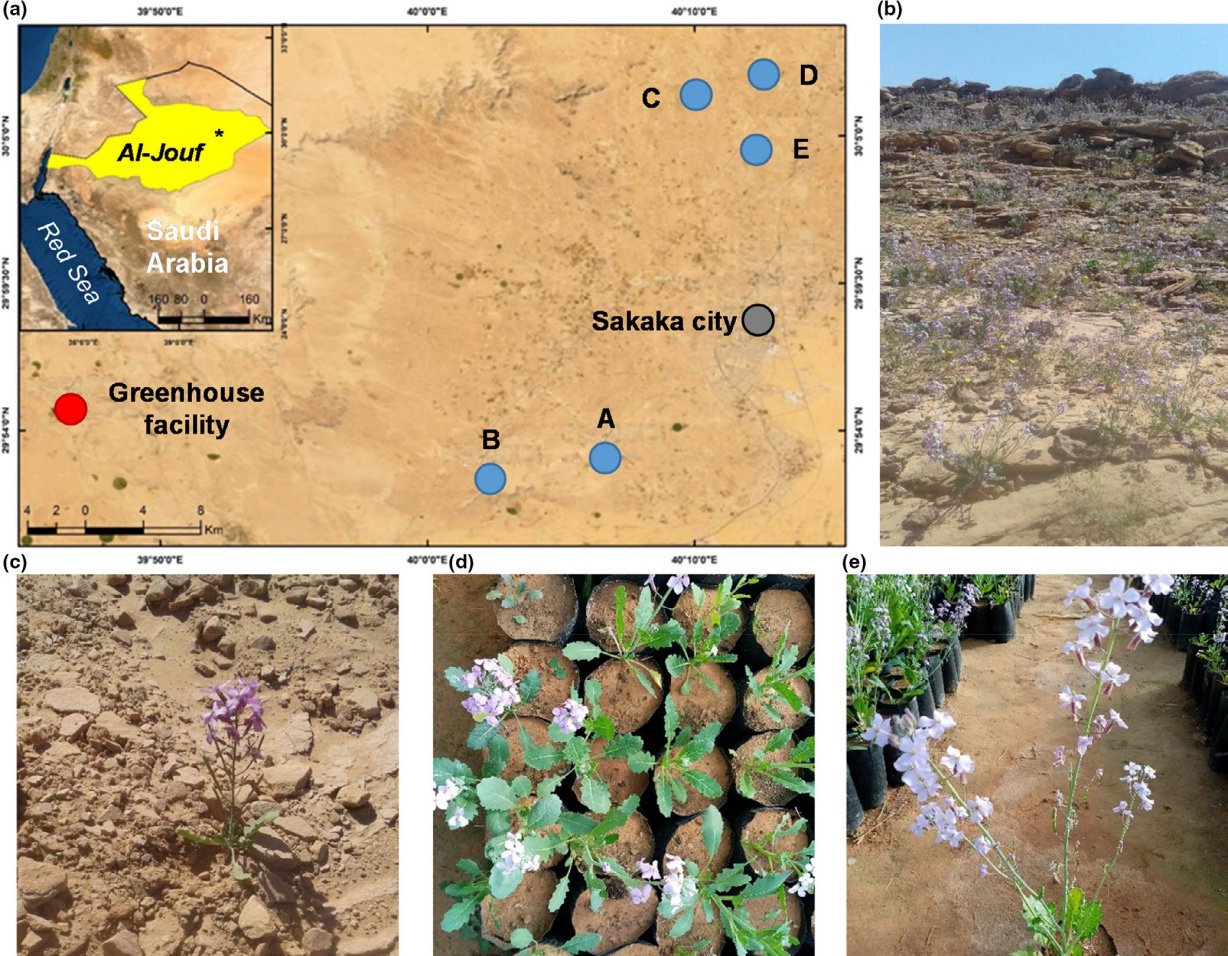
Map of the study area and photographs of *Diplotaxis acris*. (a) Location of study populations and the greenhouse facility around Sakaka city in the Al‐Jouf region in NW Saudi Arabia. The asterisk in the inner panel indicates the location of the study area in the Al‐Jouf region. (b) General view of a natural population at the flowering peak. (c) Details of a flowering individual in the wild. (d) General view of the common garden experiment with individuals at different developmental stages. (e) Details of a flowering individual in the common garden experiment

The study area is located in the Al‐Jouf region, NW Saudi Arabia (approx. 29°N–32°N, 37°E–42°E; Figure 1a). The region has a hyperarid climate with hot summers, cool winters, and scarce precipitation. Based on data from the Al‐Jouf airport meteorological station (2016–2019; Figure S1), mean total annual precipitation was 93.1 mm (range = 39.6–156.3 mm), with the rainy period extending from November to May, followed by five consecutive months of severe drought (June–October). The average total monthly precipitation during the entire rainy period for these years was 13.5 mm (range = 1.1–83.4 mm). Mean monthly minimum temperatures (annual mean ± SD = 18.7 ± 0.9°C) ranged from a low of 5°C (February 2017) to a high of 31°C (July 2017). Mean monthly maximum temperatures (annual mean ± SD = 29.2 ± 0.6°C) ranged from a low of 14°C (January 2016) to a high of 43°C (July 2017). Finally, mean monthly relative humidity (annual mean ± SD = 25.6 ± 1.0%) was rather low, varying between 12% (July and August 2017) and 50% (November 2018).

*Diplotaxis acris* (Forssk.) Boiss. (Brassicaceae) is an annual desert herb (Figure 1b,c). The species is widespread in Saudi Arabia, in particular at the northern and western areas of the country. The core of species' distribution range includes deserts of Egypt, the Palestine region, and the Arabian Peninsula (Chaudhary, 1999), but the species can also be found in deserts of Chad, Kuwait, and Libya. *Diplotaxis acris* grows in sandy and rocky soils of wadis and runnels, which collect runoff water from surrounding, more elevated areas. In the study area, seed germination occurs mainly in November with, the first rains after the long severe drought. *Diplotaxis acris* blooms in January, and fruiting spans between February and March. Flowers (1 cm long) are purple and arranged in dense racemes. Plants can produce a single flowering stalk, but they can also lose apical dominance.

We sampled 30–40 individuals (or maternal families) from five haphazardly chosen *D. acris* populations (total *N* = 176 individuals). All populations, separated by 5–30 km from each other, were located around Sakaka city, Saudi Arabia (Figure 1a). In particular, populations A (29°52′58″N, 40°6′40.7″E) and B (29°52′13″N, 40°2′23″E) were located 10–15 km SW of the city. Populations C (30°6′33.1″N, 40°10′6.6″E), D (30°7′11.6″N, 40°12′37″E), and E (30°4′33.6″N, 40°12′22.9″E) were located 10–15 km N of the city. Populations exhibited some environmental differences. For example, populations A, B, and D were located in shallow runnels; population C was located in a flat rocky area; and population E was located in a deep runnel. There were no major differences between shallow runnels, deep runnels, and flat rocky areas in terms of solar exposure and probably temperature. Nevertheless, they differed in potential water availability for plants, being higher in runnels than in flat rocky areas. Estimates of the soil moisture content during the flowering season showed that population C (flat rocky area) had the lowest soil moisture content (mean ± SE = 1.63 ± 0.11%), whereas the others located in runnels (shallow and deep) exhibited higher and similar values (range of means ± SE = 3.33 ± 0.15%–3.87 ± 0.11% in populations D and E, respectively). All populations were located at a similar altitude (621–662 m.a.s.l.), occupied a similar area (1400–1800 m^2^), and exhibited similar *D. acris* densities (1.0–1.9 individuals/m^2^).

Vegetation in hyperarid deserts is composed of widely spaced perennial shrubs, whereas annuals occupy barren shrub interspaces. Except slight differences, all five *D. acris* populations harbored a similar plant community. Major co‐occurring perennial species were *Haloxylon salicornicum* (Moq.) Bunge ex Boiss. (Chenopodiaceae), *Nauplius graveolens* (Forssk.) Wiklund (Asteraceae), and *Artemisia judaica* L. (Asteraceae). The annual plant community included *Savignya parviflora* (Delile) Webb (Brassicaceae), *Erodium laciniatum* var. *pulverulentum* (Cav.) Boiss. (Geraniaceae), *Schismus barbatus* (L.) Thell. (Poaceae), *Plantago ciliata* Desf. (Plantaginaceae), *Plantago ovata* Forssk. (Plantaginaceae), *Trigonella stellata* Forssk. (Fabaceae), and *Spergularia bocconei* (Scheele) Graebn. (Caryophyllaceae).

### 2.2 Field sampling and greenhouse experiment

In March 2019, we collected ripe seeds from haphazardly chosen individuals, separated by 2–5 m from each other, across a similar area (approx. 300 m^2^) within each population. We kept seeds in 1.5‐ml plastic tubes at room temperature (20–24°C) in darkness until the sowing day. We used mean seed weight as a covariate to control for environmental maternal effects in the statistical analyses (see below) because environmental maternal effects can be an issue when testing genetic differentiation in offspring traits (Bischoff & Müller‐Schärer, 2010). To estimate mean seed weight for each individual from each population, we weighed to the nearest 0.1 mg three batches of 100 seeds each (176 individuals × 3 batches/individual × 100 seeds/batch = 52,800 seeds) with a GR‐200 digital balance (A&D Company, Tokyo, Japan).

To estimate quantitative genetic variation in architectural and life history traits in *D. acris*, we established an experiment in November 2019 in a plastic unconditioned greenhouse (29°54′40.9″N, 39°46′41″E; 670 m.a.s.l.; Figure S2) located 25–45 km away from the study populations (Figure 1a). The mean minimum and mean maximum temperatures recorded inside the greenhouse over the duration of the experiment were of 11 and 20°C, respectively. The relative humidity inside the greenhouse varied between 33% and 48%. The experiment included 37, 40, 39, 30, and 30 individuals from populations A, B, C, D, and E, respectively. We sowed ten replicates per individual in round plastic bags (23 × 13 cm; Figure 1d). The experimental layout comprised a complete randomization of replicates from all individuals and populations within the greenhouse. In order to keep greenhouse effects under control (see below), we used a high number of replicates per individual because of the impossibility of periodic re‐arrangement of round plastic bags throughout the experiment. We filled round plastic bags with soil collected from a site near the greenhouse, which was practically the same type of sandy soil that the one found in all study populations and because of the absolute lack of vegetation, particularly *D. acris*. Each replicate contained 150 sound seeds. Thus, this experiment included 1760 experimental units (round plastic bags) and 264,000 seeds (176 individuals × 10 replicates/individual × 150 seeds/replicate). We watered plants as needed throughout the experiment to avoid hydric stress and to evaluate the full potential of *D. acris* plants growing in optimal conditions.

We estimated the proportion of germinated seeds 2 weeks after the sowing date (November 16, 2019) when we observed the maximum number of seedlings per replicate. Two weeks later, 1 month after sowing, we thinned out replicates to one juvenile plant per replicate (Figure 1d) to avoid density‐dependent and asymmetrical competition effects. We estimated flowering date as the number of days between the sowing date and the opening of the first flower. When plants were completely fruited, we measured several architectural and life history traits. These traits included the length of the main flowering stalk, the number of secondary stalks on the main stalk, the mean length of all secondary stalks, the number of caulinar leaves of the main stalk, and the mean length of the fruits on the main stalk.

In addition, we estimated fecundity by counting the number of seeds in 10 fruits per replicate (176 individuals × 10 replicates/individual × 10 fruits/replicate = 17,600 fruits) and multiplying the average number of seeds per fruit by the total number of fruits per plant. We did not detect any significant ovule abortion or seed loss during the counting. We saw some bees inside the greenhouse when flowering was quite advanced (N. H. Gomaa, pers. obs.). Nevertheless, we believe that the vast majority of seeds came from self‐fertilization. To avoid redundancies, we excluded from the analyses some traits due to strong correlation with other traits or practically no among‐individual variability. These traits included the number of stalks emerging from the base (on average 1.08 ± 0.02 main stalks per plant), the mean length of all stalks (nearly identical to the length of the main stalk; on average 71.1 cm vs. 70.4 cm), and the number of fruits (strongly correlated with the number of seeds; *r* = .99).

We terminated the experiment when all plants were completely fruited (March 23, 2020). We dug up, rinsed, and dried out all plants (70°C for 48 h) in an FED 115 oven (BINDER GmbH, Tuttlingen, Germany). Then, we estimated the aboveground biomass, the belowground biomass, and the root‐to‐shoot ratio. We used the root‐to‐shoot ratio as a measure of individual fitness as biomass‐related traits may represent a good estimate of fitness in plants (Younginger et al., 2017). We excluded fecundity to estimate fitness because it was significantly positively correlated with aboveground biomass (*N* = 176, *r* = .93, *p* < .0001) and belowground biomass (*N* = 176, *r* = .89, *p* < .0001), and significantly negatively correlated with the root‐to‐shoot ratio (*N* = 176, *r* = −.65, *p* < .0001).

### 2.3 Statistical analyses

We tested the effect of population (random factor) and individual (random factor) nested within the population on variation in architectural and life history traits of *D. acris*. Prior to analyses, we inspected the frequency distribution of variables, the variances of data, and the existence of outliers. Based on that, we did not detect greenhouse effects and did not have to exclude any replicate from the experiment. We fitted linear mixed models (LMM) for all traits, except for seed germination. For the latter, we fitted generalized linear mixed models (GLMM), using the gamma distribution as the inverse link function. We carried out these analyses with the R package lme4 (Bates et al., 2015). We included seed weight in all analyses as a covariate to consider environmental maternal effects at all times. We also checked model residuals to make sure that the major assumptions of the analyses were acceptable. We tested significances with likelihood‐ratio tests. We obtained *R* ^2^ values for each factor and variable using the “r2_nakagawa” function (Nakagawa et al., 2017).

To quantify the degree of genetic determination in our experimental conditions (i.e., the proportion of phenotypic variance accounted for by genotypic variance) for all traits, we estimated broad sense heritability (*h* ^2^) values as *h* ^2^ = *V_G_*/(*V_G_* + *V_E_*), where *V_G_* is the estimated among‐individual variance component and *V_E_* is the residual variance (Le Corre, 2005). We computed all variance components and their 95% confidence intervals using the “remlVCA” and “VCAinference” functions of the R package VCA v.1.4.3.

Finally, to determine the effects of selection upon *D. acris*, we estimated linear and quadratic selection gradients (*β* and *γ*) and selection differentials (*s* and *C*) for the major fitness‐related developmental transitions in annuals (i.e., recruitment and flowering time), using a generalized additive model‐based (GAM) characterization of the fitness function with the R package gsg (Morrissey & Sakrejda, 2013). In our case, we used the seed germination fraction as the estimate of recruitment. We conducted all selection analyses with standardized variables. Selection gradients and selection differentials were estimated from full models including linear and quadratic effects. We did not double quadratic regression coefficients and standard errors. Traditional least squares‐based regressions yielded consistent results (analyses not shown).

## 3 RESULTS

We estimated the amount of quantitative variation in architectural and life history traits of 176 *D. acris* individuals from five populations from the hyperarid Arabian Desert by undertaking a greenhouse experiment. Overall, *D. acris* individuals exhibited very low germination rates (on average 5%; Table 1). Plants produced the first flower in slightly more than 2 months since seed sowing, with flowering times spanning 25 days between the earliest and the latest individual (Table 1). Plants reached a considerable size in the greenhouse (Figure 1d,e) with tall main stalks (on average >70 cm; Table 1) and long secondary stalks (on average ca. 45 cm; Table 1). Individual plants produced hundreds of fruits (on average ca. 115 fruits) and thousands of seeds (on average ca. 18,000 seeds; Table 1).

**TABLE 1:** Linear mixed models or generalized linear mixed models testing the effect of population and individual nested within population on architectural and life history traits of *Diplotaxis acris*

Traits	Covariate	Population		Individual		Mean ± SE	Range among individuals
	*t*‐value	*χ* ^2^	*R* ^2^	*χ* ^2^	*R* ^2^		
Length apical flowering stalk	−0.25 *ns*	5.31*	.11	466.01***	.62	71.05 ± 1.28	36.50–109.30 cm
No. secondary stalks	1.36 *ns*	13.96***	.17	284.45***	.47	2.61 ± 0.07	1.10–6.22 stalks
Mean length secondary stalks	0.47 *ns*	6.38*	.13	464.75***	.65	44.47 ± 1.19	11.45–80.08 cm
No. leaves apical flowering stalk	0.73 *ns*	0.01 *ns*	.00	358.22***	.69	8.57 ± 0.28	3.30–21.40 leaves
Mean fruit length	−0.28 *ns*	7.57**	.12	429.83***	.61	4.38 ± 0.04	3.06–5.90 cm
Aboveground biomass	1.35 *ns*	7.12**	.14	458.35***	.63	6.49 ± 0.25	1.52–21.72 g
Belowground biomass	1.48 *ns*	19.61***	.19	388.54***	.59	0.65 ± 0.02	0.02–0.13 g
Root‐to‐shoot ratio	−0.43 *ns*	0.01 *ns*	.00	127.01***	.23	0.11 ± 0.001	0.001–0.07
Seed weight	–	9.90**	.09	1333.10***	.90	1.30 ± 0.02 × 10^−2^	0.70–2.50 ×10^−2^ g
Seed germination	1.08 *ns*	21.29***	.08	533.68***	.43	0.05 ± 0.003	0.01–0.24 (proportion)
Flowering time	−2.27*	10.46**	.05	15.58***	.36	70.02 ± 0.33	57.80–82.78 days
Fecundity	1.51 *ns*	11.01***	.14	580.12***	.70	17.98 ± 0.92 × 10^3^	3.96–58.93 ×10^3^ seeds

Note

Seed weight was used as a covariate in all analyses. Factors were tested through likelihood‐ratio tests. The proportion of variance explained by each factor and variable are given. The mean (±SE) and the range of values among individuals (*N* = 176) for each trait are also provided. Asterisks indicate significance: ***p < .001, **p < .01, *p < .05, *ns*; nonsignificant.

**FIGURE 2:**
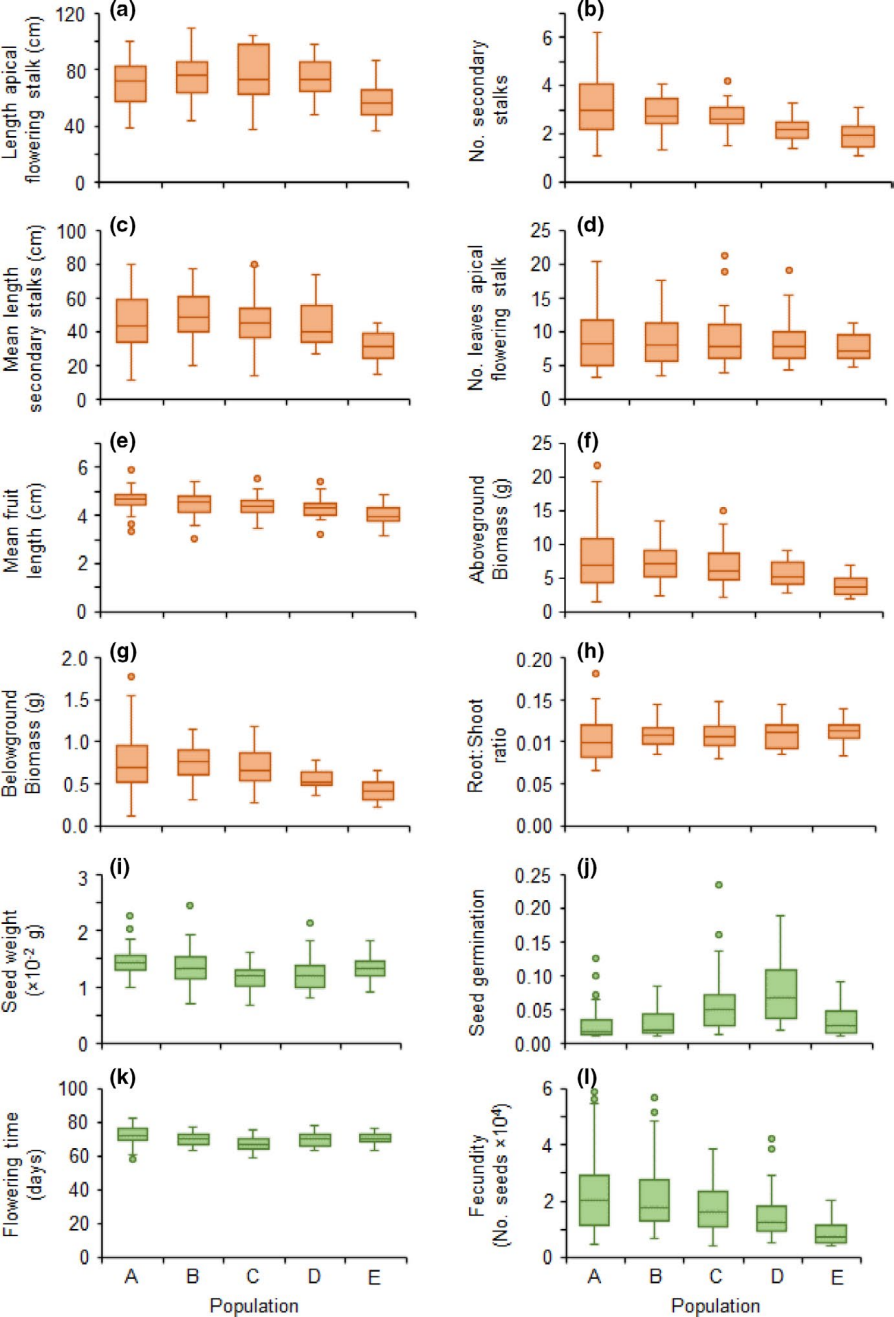
Summary statistics for architectural (orange) and life history (green) traits of *Diplotaxis acris* populations. Boxes show the lower and upper quartiles; whiskers indicate the minimum and maximum values; and the line is the median of observations and dots indicate atypical values

We used linear models to test the random effects of population and individual nested within populations, using seed weight as a covariate, on architectural and life history traits. We found that seed weight had little effect on variation in all traits, except for flowering time (Table 1), indicating that seed weight affected flowering time differently among populations. In fact, the relationship between seed weight and flowering time was negative (range of *r* = −.37 to −.06; i.e., plants with heavier seeds flowered earlier) but only significant in one population (*N* = 30, *r* = −.37, *p* = .047; population D). Overall, linear models indicated that variation among individuals mostly accounted for variation in both architectural and life history traits (Table 1). Although population was also significant for most traits (Table 1 and Figure 2), except for the number of leaves on the main flowering stalk and belowground biomass, the variation explained by populations was substantially lower (range *R* ^2^ = .00–.19) than that explained by individuals (range *R* ^2^ = .23–.90; Table 1).

We also explored trait–trait correlations to illustrate the configuration of architectural and life history traits in *D. acris*. Given the low amount of variation explained by population, we focused on the results from the correlation pooling all individuals from all populations. All architectural traits exhibited strong positive correlations among them (Table 2). Architectural traits also showed strong positive correlations with fecundity (i.e., larger plants produced more seeds; Table 2). The negative correlation of architectural traits with the root‐to‐shoot ratio was the result of the way in which this variable was estimated. Interestingly, one architectural trait and one life history trait (the number of secondary stalks and seed weight) were significantly positively correlated (*r* = .16, *p* = .032; Table 2), showing a trend for plants with heavier seeds having a more vigorous branching pattern. Finally, two life history traits (seed germination and flowering time) were significantly negatively correlated (*r* = −.35, *p* < .0001; Table 2), indicating that plants with higher germinability flowered earlier.

**TABLE 2:** Trait–trait correlations for *Diplotaxis acris* individuals from all populations (*N* = 176)

	LAFS	NSS	MLSS	NLAFS	MFL	AB	BB	RSR	SW	SG	FT	FEC
LAFS	–	***	***	***	***	***	***	***	*ns*	*ns*	*ns*	***
NSS	**0.60**	–	***	***	***	***	***	***	*	*ns*	*ns*	***
MLSS	**0.91**	**0.68**	–	***	***	***	***	***	*ns*	*ns*	*ns*	***
NLAFS	**0.56**	**0.62**	**0.65**	–	***	***	***	***	*ns*	*ns*	*ns*	***
MFL	**0.55**	**0.48**	**0.60**	**0.46**	–	***	***	***	*ns*	*ns*	*ns*	***
AB	**0.81**	**0.89**	**0.90**	**0.70**	**0.56**	–	***	***	*ns*	*ns*	*ns*	***
BB	**0.81**	**0.89**	**0.89**	**0.67**	**0.55**	**0.97**	–	***	*ns*	*ns*	*ns*	***
RSR	**−0.64**	**−0.57**	**−0.69**	**−0.54**	**−0.46**	**−0.69**	**−0.55**	–	*ns*	*ns*	*ns*	***
SW	−0.01	**0.16**	0.05	0.04	0.07	0.12	0.14	0.01	–	*ns*	*ns*	*ns*
SG	0.04	−0.09	−0.01	0.07	−0.02	−0.08	−0.10	−0.06	−0.04	–	***	*ns*
FT	−0.13	0.02	−0.14	0.02	0.06	−0.06	−0.07	0.08	−0.02	**−0.35**	–	*ns*
FEC	**0.73**	**0.81**	**0.84**	**0.77**	**0.61**	**0.93**	**0.89**	**−0.65**	0.13	−0.05	−0.04	–

Note

Architectural traits are length apical flowering stalk (LAFS), no. secondary stalks (NSS), mean length secondary stalks (MLSS), no. leaves apical flowering stalk (NLAFS), mean fruit length (MFL), aboveground biomass (AB), belowground biomass (BB), and root‐to‐shoot ratio (RSR). Life history traits are seed weight (SW), seed germination (SG), flowering time (FT), and fecundity (FEC). All significant correlations are in bold. Asterisks indicate significance: ***p < .001, **p < .01, *p < .05, *ns*; nonsignificant.

To evaluate the degree of genetic determination of architectural and life history traits in *D. acris*, we estimated broad sense heritability (*h* ^2^) values for all traits. All *h* ^2^ values were high (Table 3), as expected by the variation in traits explained by individuals in linear models (Table 1). Seed weight and fecundity, two life history traits, exhibited the highest *h* ^2^ values (Table 3). All architectural traits, except the root‐to‐shoot ratio, showed very similar *h* ^2^ values (range of *h* ^2^ = 0.64–0.78 for the number of secondary stalks and the mean length of secondary stalks, respectively). The lowest *h* ^2^ values were for the root‐to‐shoot ratio and flowering time (Table 3). We recorded almost all the lowest *h* ^2^ values in population E, whereas populations A and D exhibited the largest *h* ^2^ values for many traits (Table 3).

**TABLE 3:** Broad sense heritability (*h* ^2^) for architectural and life history traits of *Diplotaxis acris*

Traits	All populations	Range among populations
	*h* ^2^	*h* ^2^
Length apical flowering stalk	0.715 (0.678–0.740)	0.459 (E)–0.799 (C)
No. secondary stalks	0.639 (0.596–0.668)	0.162 (E)–0.821 (A)
Mean length secondary stalks	0.778 (0.747–0.799)	0.415 (E)–0.846 (C)
No. leaves apical flowering stalk	0.690 (0.650–0.716)	0.139 (E)–0.794 (A)
Mean fruit length	0.731 (0.695–0.755)	0.380 (E)–0.811 (B)
Aboveground biomass	0.767 (0.734–0.788)	0.322 (E)–0.811 (C)
Belowground biomass	0.770 (0.737–0.791)	0.402 (E)–0.803 (A)
Root‐to‐shoot ratio	0.367 (0.318–0.402)	0.070 (E)–0.468 (A)
Seed weight	0.989 (0.987–0.989)	0.980 (A)–0.993 (D)
Seed germination	0.773 (0.722–0.769)	0.556 (E)–0.750 (C)
Flowering time	0.416 (0.367–0.451)	0.223 (E)–0.548 (A)
Fecundity	0.834 (0.798–0.851)	0.571 (E)–0.858 (D)

Note

Mean (95% CI) values are given for estimates using all populations. The range of *h* ^2^ values among populations are also given, indicating the population in parenthesis.

Finally, we estimated linear and quadratic selection gradients (*β* and *γ*) and selection differentials (*s* and *C*) for recruitment (given by the seed germination fraction) and flowering time with a GAM‐based approach to evaluate how natural selection acted upon *D. acris* individuals. Despite some marginal significances, the results showed that neither recruitment nor flowering time significantly contributed to fitness, estimated by the root‐to‐shoot ratio (Table 4) in our experimental conditions. We found the same results when using fecundity, aboveground biomass or belowground biomass as estimates of fitness (results not shown).

**TABLE 4:** Linear and quadratic selection gradients (*β* and *γ*) and selection differentials (*s* and *C*) for recruitment and flowering time using a GAM‐based approach for *Diplotaxis acris* for each population

Population		Linear			Quadratic		
		Recruitment	Flowering time		Recruitment	Flowering time	Interaction
A (*N* = 37)	*β*	2.850 (1.676) *ns*	−0.005 (0.149) *ns*	*γ*	−3.987 (2.58) *ns*	−0.027 (0.265) *ns*	−1.570 (1.883) *ns*
	*s*	−0.007 (0.071) *ns*	−0.067 (0.080) *ns*	*C*	0.085 (0.149) *ns*	0.105 (0.147) *ns*	0.040 (0.072) *ns*
B (*N* = 40)	*β*	0.107 (0.190) *ns*	−0.115 (0.083) *ns*	*γ*	0.445 (0.977) *ns*	−0.152 (0.168) *ns*	−0.105 (0.224) *ns*
	*s*	0.144 (0.085) *ns*	−0.106 (0.085) *ns*	*C*	0.229 (0.165) *ns*	−0.114 (0.059) *ns*	−0.081 (0.055) *ns*
C (*N* = 39)	*β*	−0.132 (0.203) *ns*	0.064 (0.117) *ns*	*γ*	0.153 (0.601) *ns*	0.359 (0.364) *ns*	0.252 (0.325) *ns*
	*s*	−0.110 (0.055)*	0.040 (0.061) *ns*	*C*	−0.096 (0.096) *ns*	−0.032 (0.082) *ns*	0.093 (0.058) *ns*
D (*N* = 30)	*β*	0.270 (0.206) *ns*	0.264 (0.209) *ns*	*γ*	−0.222 (0.815) *ns*	−0.052 (0.715) *ns*	0.280 (0.405) *ns*
	*s*	0.096 (0.076) *ns*	0.047 (0.083) *ns*	*C*	−0.036 (0.082) *ns*	0.019 (0.086) *ns*	0.038 (0.058) *ns*
E (*N* = 30)	*β*	−0.026 (0.291) *ns*	−0.007 (0.130) *ns*	*γ*	0.000 (1.758) *ns*	−0.004 (0.393) *ns*	0.179 (0.474) *ns*
	*s*	−0.053 (0.081) *ns*	−0.029 (0.074) *ns*	*C*	−0.148 (0.142) *ns*	−0.060 (0.078) *ns*	0.143 (0.062)*
All (*N* = 176)	*β*	−0.013 (0.127) *ns*	−0.025 (0.044) *ns*	*γ*	0.000 (0.508) *ns*	0.007 (0.049) *ns*	0.066 (0.098) *ns*
	*s*	−0.033 (0.036) *ns*	−0.020 (0.039) *ns*	*C*	−0.057 (0.057) *ns*	0.035 (0.062) *ns*	0.072 (0.036)*

Note

Results for all populations are also given. The number of individuals per year is indicated within parentheses next to the population code. Asterisks indicate significance: ***p < .001, **p < .01, *p < .05, *ns*; nonsignificant.

## 4 DISCUSSION

Depicting the phenotypic space enables us to comprehend the evolutionary pathways that organisms can take to increase performance and long‐term survival in their changing environments. In this study, we focused on phenotypic space structure of the annual desert plant *D. acris* thriving in hyperarid deserts, which are the harshest end of the environmental gradient found in drylands. Given the current accelerated environmental changes at a global unprecedented scale (Loarie et al., 2009; Neukom et al., 2019) and the expected expansion of drylands with global warming (Feng & Fu, 2013), several annual plant species might already be experiencing harsher environmental conditions and perhaps displacements of their phenotypic spaces toward new phenotypic configurations. Thus, *D. acris* exemplifies a successful evolutionary history of adaptation to hot, dry and markedly seasonal environments, which points to one of the possible evolutionary pathways to follow as the vast terrestrial land surface occupied by drylands becomes warmer and drier.

To determine the extent of the phenotypic space in *D. acris*, we conducted a greenhouse experiment and quantified the genetic component of phenotypic variation in architectural and life history traits of 176 *D. acris* individuals from five Saudi populations. The results showed that most of the variance was explained by individuals within populations, whereas populations accounted for up to 83% less variance for both architectural and life history traits (Table 1). These results suggest that the hyperarid environmental conditions in which *D. acris* completes the life cycle constraint population divergence, a process that seems to be more intense in life history traits than in architectural traits (Table 1). We believe that regional‐scale hyperarid environmental conditions tend to minimize large‐scale spatial heterogeneity, which is known to intensify spatial patterns by enhancing population dynamics (Getzin et al., 2008) and promoting phenotypic plasticity (Lázaro‐Nogal et al., 2015) in plants. Although we cannot delimit the extent of such regional environmental homogeneity affecting population divergence in *D. acris*, significant variation in water availability and annual precipitation are likely to be the main factors, accounting for among‐population variation in key biological processes, such as flowering phenology and seed bank dynamics, as shown for other plant species from hyperarid deserts (Gomaa, 2019).

Our experiment did not allow the identification of the ecological sources accounting for among‐individual variation. Nevertheless, we hypothesize that *D. acris* could be experiencing the effects of fine‐scale environmental heterogeneity on genetic differentiation of phenotypic traits, as commonly observed in several plant species (Argyres & Schmitt, 1991; Galloway, 1995; Kalisz, 1986; Mitchell‐Olds & Bergelson, 1990; Prati & Schmid, 2000; Schemske, 1984; Stratton, 1994; Stratton & Bennington, 1996). Given the rigor of hyperarid environments, one might expect strong directional selection to decrease genetic variance in phenotypic traits (Blows & Hoffmann, 2005). However, this seems not to be happening in *D. acris*. Several processes may account for the among‐individual genetic variation in architectural and life history traits in *D. acris* as well as for the apparent viability of study populations. For example, it has been shown that even in annuals with a high selfing ability, one admixture event suffices to yield rapid changes in phenotypic variation (Palacio‐Lopez & Molofsky, 2021). Hence, the potential of generating novel genetic variants is more than relevant in every generation. Furthermore, environmental heterogeneity, regardless of its scale, can also dramatically influence selection on fitness‐related traits (Exposito‐Alonso et al., 2018; Palacio‐Lopez et al., 2020) in a relatively short time frame. In fact, fast genetically based evolutionary changes imposed by environmental changes (i.e., rapid evolution) has been quantified in annuals over just a few generations (Etterson et al., 2016; Frachon et al., 2017; Franks & Hoffmann, 2012; Franks et al., 2007; Gómez et al., 2018; Maron et al., 2004; Rhoné et al., 2010; Sultan et al., 2013). This phenomenon strongly promotes within‐population genetic variation in demographically viable populations.

Interestingly, our selection analysis did not detect any significant effect of the two most important developmental stages in annual plants, that is, recruitment and flowering time, on fitness (Table 4). This may have two interpretations. On the one hand, the first explanation may be methodological. Given that we thinned out pots to one single plant per replicate, we could neither estimate actual survival (e.g., the proportion of seedlings becoming reproductive plants) nor fitness as the product between survival and fecundity (Exposito‐Alonso et al., 2018). Instead, we used the root‐to‐shoot ratio based on the known value of biomass‐related traits as fitness estimates (Younginger et al., 2017). Clearly, we need further experiments undertaken in natural settings to estimate among‐individual variation in survival rates in *D. acris* and to determine the role of developmental transitions in fitness. Such experiments should consider the among‐individual genetic relatedness, which is always a strong predictor of plant performance (Stachowicz et al., 2013) and a factor accounting for plant population divergence (Castilla et al., 2020; Marcer et al., 2016).

On the other hand, the other nonexclusive explanation may be biological. Given the severity of hyperarid environments, and the fact that the study region falls in the species' distribution core, we might be dealing with fine‐tuned phenotypes to their environments. Environmentally induced phenotypic fine‐tuning has been quantified in other annuals, such as *Arabidopsis thaliana*, which increases seed dormancy and advances flowering time as the environment becomes drier and more seasonal (Debieu et al., 2013; Marcer et al., 2018; Vidigal et al., 2016). Hence, if *D. acris* were well‐adapted to their demanding hyperarid environments, the extent of maladaptation, and its subsequent reductions in fitness (Brady et al., 2019) would be limited, as suggested by our selection analysis. Thus, *D. acris* populations would mostly be subject to inherent demographic oscillations, probably determined by the timing and amount of annual rainfall. Nevertheless, it is hard to believe that *D. acris* individuals do not exhibit variation in performance and fitness in their natural environments, but such variation might be constrained by the extreme environmental conditions in which *D. acris* individuals complete the life cycle. In this hypothetical scenario, phenotypic plasticity would acquire a relevant role to increase plant performance among individuals experiencing small‐scale environmental heterogeneity (Lázaro‐Nogal et al., 2015). Other studies in the annual *A*. *thaliana* also indicated that populations from warmer environments exhibited higher plasticity that those from cooler environments (Exposito‐Alonso et al., 2018). Given the dramatic differences in size between plants growing in natural populations and greenhouse conditions (Figure 1c,e), it is clear that the plastic ability of *D. acris* is more than relevant. Further experiments are also required to quantify among‐individual variation in phenotypic plasticity and its neutral or adaptive nature in *D. acris*.

Finally, we can state that *D. acris* exhibited an integrated phenotype, particularly for architectural traits, which showed strong correlations among them and were strong predictors of fecundity (Table 2). In contrast, as far as life history traits are concerned, only seed germination tended to be negatively correlated with flowering time, stressing the evolutionary importance of completing the life cycle as fast as possible in those *D. acris* individuals with higher germinability. Interestingly, fecundity did not show significant correlations with any life history trait (Table 2). In other words, within the window of opportunity for completing the life cycle successfully, *D. acris* individuals invest all the resources to produce as many seeds as possible enhanced by the architectural trait integration. Nevertheless, seed quality also matters in *D. acris* because heavier seeds showed a trend for earlier flowering and a more vigorous branching pattern, which led to higher fecundity.

Overall, we conclude that *D. acris*, a specialist in hyperarid deserts, can provide useful hints of the effects of increasing warming and aridity on the phenotypic space structure in several annual plants species is broad terrestrial regions. We are well‐aware of the important value of standing genetic variation to persist in changing environments (Barrett & Schluter, 2008; Exposito‐Alonso et al., 2018; Jump et al., 2009; Matuszewski et al., 2015). Furthermore, we also know how fast annual plant populations can change their genetic composition in just a few years due to selection and/or demography (Gómez et al., 2018; Kuester et al., 2016; Nevo et al., 2012; Van Dijk & Hautekèete, 2014; Vigouroux et al., 2011). On top of that, harsher environments might promote higher phenotypic plasticity (Exposito‐Alonso et al., 2018; Lázaro‐Nogal et al., 2015; Matesanz et al., 2020) and tighter phenotypic integration (Benavides et al., 2021; Dwyer & Laughlin, 2017; Michelaki et al., 2019). For all these reasons, we believe that most annuals will have a chance to thrive in a warmer, more arid, and more seasonal environments by shifting their phenotypic spaces toward new scenarios in which phenotypic integration and seed‐related traits will acquire a higher relevance. The pace and heterogeneity of increasing warming and aridity across the vast land surface occupied by drylands will determine the eventual outcome for several annual plant species in the coming decades.

## AUTHOR CONTRIBUTIONS

**Nasr H. Gomaa:** Conceptualization (equal); Formal analysis (supporting); Investigation (equal); Methodology (equal); Resources (lead); Validation (equal); Writing‐original draft (supporting). **F. Xavier Picó:** Conceptualization (equal); Formal analysis (lead); Investigation (equal); Methodology (equal); Resources (supporting); Validation (equal); Writing‐original draft (lead).

## PERMIT

No permits were required.

## Supporting information

Figure S1‐S2

## Data Availability

Data deposited in the Dryad repository: https://doi.org/10.5061/dryad.f4qrfj6wx.
