# Supplementary material for: Depicting the phenotypic space of the annual plant Diplotaxis acris in hyperarid deserts
Source: Ecol Evol. 2021 Nov 5;11(22):15708–19. doi: 10.1002/ece3.8232 (PMC8601918; doi:10.1002/ece3.8232)

**Supporting information**

Figure S1. Mean monthly maximum temperature (red line), mean monthly minimum temperature (blue line), total monthly precipitation (green bars) and mean monthly relative humidity (pink bars) in the region of the Arabian Desert. Data from the Al-Jouf airport meteorological station (2016-2019).


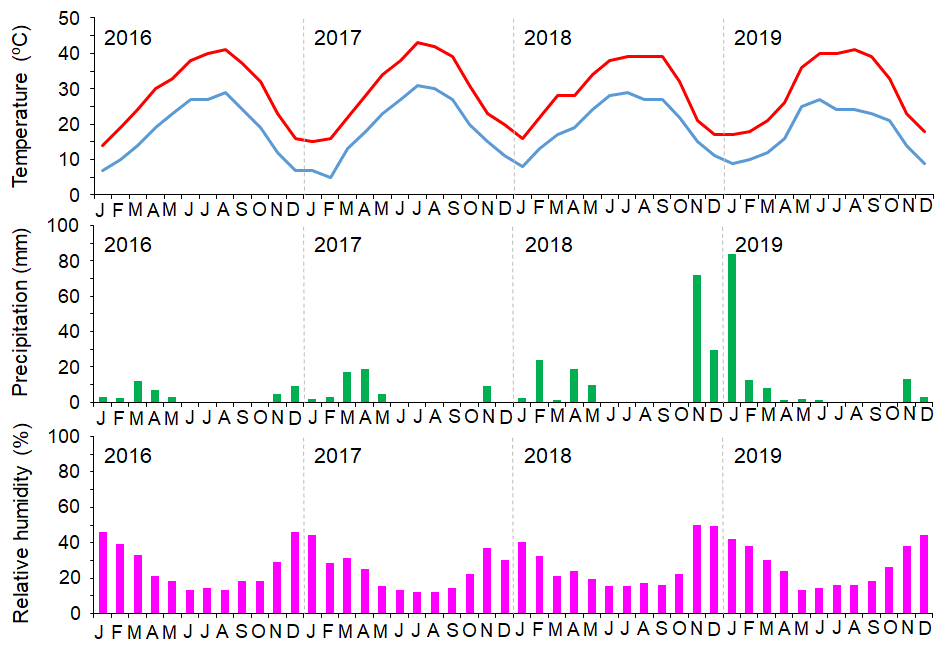


Figure S2. Views of the unconditioned greenhouse and *D. acris* plants during the flowering and fruiting period.


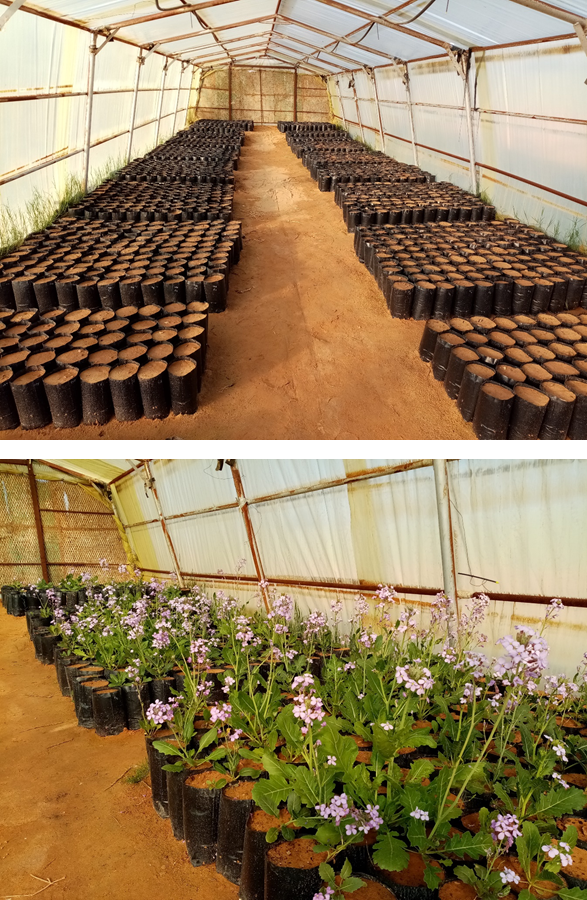

Supplement: Supplementary file 1 — Figure S1‐S2 [file ECE3-11-15708-s001.docx]
